# Advancing Sustainability: Geraniol-Enhanced Waterborne Acrylic Pressure-Sensitive Adhesives without Chemical Modification

**DOI:** 10.3390/ma17204957

**Published:** 2024-10-10

**Authors:** Ludovica Di Lorenzo, Simone Bordignon, Michele R. Chierotti, Ignazio Andrea Alfeo, Adrian Krzysztof Antosik, Valentina Brunella

**Affiliations:** 1 Department of Chemistry and NIS Centre, University of Turin, Via Pietro Giuria 7, 10125 Torino, Italy; ludovica.dilorenzo@unito.it (L.D.L.); simone.bordignon@unito.it (S.B.); michele.chierotti@unito.it (M.R.C.); 2 Centro Ricerche Fiat SCPA (CRF), Stellantis, 10135 Turin, Italy; ignazioandrea.alfeo@crf.it; 3 Department of Chemical Organic Technology and Polymeric Materials, Faculty of Chemical Technology and Engineering, West Pomeranian University of Technology, Pulaskiego 10, 70-322 Szczecin, Poland; adriankrzysztofantosik@gmail.com

**Keywords:** acrylic PSA, bio-based adhesive, emulsion polymerization, green copolymers

## Abstract

The escalating global emphasis on sustainability, coupled with stringent regulatory frameworks, has spurred the quest for environmentally viable alternatives to petroleum-derived materials. Within this context, the adhesives industry has been actively seeking renewable options and eco-friendly synthesis pathways. This study introduces geraniol, a monoterpenoid alcohol, in its unmodified form, as a key component in the production of waterborne pressure-sensitive adhesives (PSAs) based on acrylic latex through emulsion polymerization. Multiple formulations were developed at varying reaction times. The adhesives underwent comprehensive chemical characterization employing techniques such as Fourier-transform infrared spectroscopy (FTIR), thermogravimetric analysis (TGA), differential scanning calorimetry (DSC), Nuclear Magnetic Resonance (NMR), Gel Permeation Chromatography (GPC), and dynamic light scattering (DLS). The viscosities of the formulations were measured between 4000 and 5000 cP. Adhesion tests showed peel strength values of 0.52 N/mm on cardboard and 0.32 N/mm on painted steel for the geraniol-based formulations. The results demonstrate the potential for geraniol-based PSAs to offer a sustainable alternative to petroleum-derived adhesives, with promising thermal and adhesive properties.

## 1. Introduction

Since the late 19th century, pressure-sensitive adhesives (PSAs) have been widely used in various scientific, industrial, and everyday applications, such as packaging, labels, sticky notes, and plastic wraps [1]. The global PSA market is experiencing rapid growth, with an expected market value of $16.9 billion by 2027 [2]. The success of this class of adhesives is attributed to their ability to be applied to any type of substrate without any chemical reaction, thanks to the evaporation of solvents and very low pressure [3]. Typically, they establish weak bonds with the substrate when applied with gentle pressure, usually by hand. Specifically, adhesion results from Van der Waals and polar forces between the adhesive and the substrate, making the surface cleaning of the substrate crucial when using this type of adhesive [4]. All of these characteristics make them easy and safe to use. There are four main categories of PSAs: natural rubber, synthetic rubber, silicone, and acrylic. Acrylic adhesives are the most common ones, because of their good resistance to oxidation, high transparency, color stability, and low glass transition temperature (Tg), typically ranging from −20 °C to −70 °C [5]. They also exhibit resistance to high temperatures, they offer specialized formulations with tailored properties, and are more cost-effective than silicone PSAs [6,7]. Commonly used acrylate monomers include n-butyl acrylate (BA), methyl acrylate, methyl methacrylate (MMA), and/or 2-ethylhexyl acrylate (2-EHA), copolymerized using various polymerization processes such as emulsion polymerization, bulk polymerization, and solvent polymerization. Emulsion polymerization offers efficient heat removal, controls the submicron particle size, and maintains low viscosity at high polymer concentrations. It also supports higher polymerization rates and produces higher-molecular-weight polymers, while reducing volatile organic compound (VOC) emissions by using aqueous dispersions, addressing environmental concerns [8,9,10]. Acrylic PSAs typically consist of 70–90 wt.% of soft monomers, characterized by low Tg, such as BA (Tg = −54 °C), and 30–10 wt.% of hard monomers, characterized by high Tg, like MMA (Tg = 105 °C). The soft monomers contribute to the adhesiveness and tackiness of the adhesive, while the hard monomers enhance the cohesion. Typically, a small amount of an unsaturated carboxylic acid is also included to improve wettability and to enhance resistance to peel and shear [11,12,13]. However, commercial acrylic PSAs rely heavily on finite fossil resources and sometimes involve the use of solvents, leading to the production of VOCs. This dependence creates significant environmental challenges in today’s environmentally conscious society. Additionally, global regulations have been becoming increasingly stringent, driving the development of more environmentally friendly alternatives to oil-based materials [4,5,14,15]. The interest in the production of bio-based adhesives is not entirely new in the adhesives industry. The inspiration for using natural biopolymers as adhesives comes from observing nature’s adhesive systems, such as the sticky glues produced by plants or small animals for self-defense. However, natural adhesives are typically used in their virgin state and have inherent drawbacks related to their chemical nature, including weak UV resistance, low water resistance, and variability in composition. Only highly optimized renewable products can compete with their petrochemical counterparts, contributing to the success of bio-economic development. In recent years, several studies on new and more renewable building blocks for PSAs have been published. Imamet et al. [16] and Vendamme et al. [10] have provided a comprehensive overview with examples of fatty acids [17,18], starch-derived [19,20] building blocks, or their combinations [21]. Terpenes, a diverse class of renewable organic compounds with a wide range of structural and functional variations [22,23,24,25,26], have shown promise for the PSA bio-sourced market. Their easy modification into reactive monomers has made their incorporation successful in various applications, including coatings [27], resins [28], and thermoplastic elastomers [29]. Sun et al. [30], aiming to enhance characteristics such as thermal stability, peeling resistance, and transparency for PSAs designed for the opto-electronic market, explored a new approach using bio-based PSAs derived from soybean oil. Badía et al. [12,31] synthesized PSAs using 72% bio-sourced materials through emulsion or latex polymerization. They used 2-octyl acrylate (2OA, 73% bio-sourced) derived from castor oil and isobornyl methacrylate (IBOMA, 71% bio-sourced), which is in turn derived from pine resin. They also produced a latex coating containing 2OA and IBOMA along with a sugar-based vinyl monomer (EcoMer, EcoSynthetix) to introduce a biodegradable element into their formulation [4,32,33]. Furthermore, Cheng Fang et al. [34] sought to replace the traditional petroleum-based hard monomer MMA in waterborne PSA formulations with IBOMA. Their work demonstrated that the introduction of IBOMA led to a higher storage modulus (Gʹ) and shear strength, but lower loop tack and peel strength compared to counterparts prepared with MMA [34]. Tetrahydrogeraniol acrylate (THGA), derived from geraniol, is another monomer that has emerged as a robust substitute for BA and/or 2-EHA in the design of thermoplastic elastomers and acrylic PSAs [4,5,34,35]. It is worth noting that the research mentioned above primarily focuses on the use of biological sources (especially terpenes) after chemical functionalization.

The objective of this study was to partially replace soft monomer BA in waterborne acrylic PSA formulations with pure geraniol (the trans-isomer of 3,7-dimethyl-2,6-octadien-1-ol) without any chemical functionalization. Considering the lengthy reaction cycles and challenging purification phases required to obtain this type of product, additional synthetic steps would increase costs and reduce environmental sustainability. In this study, green latex containing geraniol was synthesized using a monomer-starved seeded semi-continuous emulsion polymerization process at different reaction times to evaluate the most suitable time for these products. This type of emulsion polymerization is initially conducted to prepare monodisperse polymer particles. A portion of the polymer particle dispersion is then used as a seed solution for subsequent seeded emulsion polymerization [36]. The thermal properties, viscosity, and particle size of the latex were investigated to study the influence of bio-sourced geraniol on the latex. Additionally, FTIR and peeling tests were employed to characterize the chemical structure and adhesive properties of the green latexes, respectively. The results were compared with those of oil-based acrylic PSAs.

## 2. Materials and Methods

Butyl acrylate—BuA (Sigma-Aldrich, St. Louis, MO, USA, +99%), methyl methacrylate—MMA (Sigma-Aldrich, +99%), acrylic acid—AA (Sigma-Aldrich, +99%), hydroxyethyl methacrylate—HEMA (Sigma-Aldrich, +99%), and geraniol—GER (Sigma-Aldrich, +98%) were used as received without any further purification. Sodium dodecyl sulphate—SDS (Sigma-Aldrich) was used as surfactant and potassium persulfate—KPS (Sigma-Aldrich, +99%) and NaHCO_3_ (commercial) were used as initiator and buffer, respectively. Deionized water was used as a solvent during this study.

### 2.1. Emulsion Polymerization Procedure

All polymers (Table 1) were prepared at 52–55% of solid content; to keep this value constant, water was added. Below (Table 2), the typical experiment: 60 mL of BA, 15 mL of geraniol, 10 mL of MMA, 0.8 mL of AA, 1.5 mL of HEMA, 1.22 g of SDS, and 20 mL of deionized water were mixed in a 250 mL round-bottom flask and stirred vigorously at 500 rpm to form the pre-emulsion. Another 250 mL two-neck round-bottom flask with a reflux condenser was filled with 0.26 g of NaHCO_3_, 5 g of pre-emulsion, 0.22 g of KPS, and 40 mL of deionized water with a stirring rate of 270 rpm at 73 °C and kept at this temperature for 30 min. Then, a KPS aqueous solution was prepared with 0.30 g of KPS in 19 mL of water; this one and the residual pre-emulsion one were added dropwise into the reacting mixture for 4 h (PSA 1), 5 h (PSA 2), and 6 h (PSA 3) under stirring at 500 rpm. After this, the reaction was allowed to proceed for an additional 1 h to increase the monomer conversion. The latex was cooled down to room temperature. While no purification step followed the reaction of PSA 1 and PSA 2, PSA 3 was subjected to two different treatments. One variant of PSA 3 was analyzed without any purification steps, while another variant, namely *PSA 3, underwent a purification process in which the copolymer was precipitated from water by the addition of methanol. After decanting the methanolic solution, the purified polymer was washed twice with fresh methanol to remove any residual unreacted monomer, free emulsifier, and initiator, ensuring the complete purification of the sample. The washing process was carried out using a centrifuge; the polymer–methanol mixture was centrifuged at 4000–6500 rpm for 10–30 min at room temperature. Following centrifugation, the methanol was discarded, and the process was repeated to ensure thorough purification before proceeding to the application and drying stage.

The decision to incorporate 20 wt.% geraniol into the acrylic PSAs was driven by the aim to achieve a harmonious balance between enhancing the organic green content and preserving the adhesive performance. This specific weight percentage is calculated solely based on the monomer content as 100% of the weight.

### 2.2. Latex Characterization

#### 2.2.1. Nuclear Magnetic Resonance

The ^1^H and ^13^C solution (CDCl_3_) NMR spectra were acquired on a Jeol ECZR 600 instrument (JEOL Ltd., Tokyo, Japan), operating at 600.17 and 150.91 MHz for ^1^H and ^13^C nuclei, respectively.

For the ^1^H spectra, 128 scans were collected for each sample, employing a relaxation delay of 5 s; as for ^13^C NMR analyses, for each sample, 10,000 scans were acquired, with a relaxation delay of 4.5 s.

#### 2.2.2. Fourier-Transform Infrared Spectroscopy

Fourier-transform infrared (FTIR) spectra were recorded on a Perkin Elmer Spectrum (PerkinElmer, Inc., Waltham, MA, USA) 100 in the attenuated total reflectance (ATR) mode with a diamond crystal, using 16 scans per spectrum and a resolution of 4 cm^−1^ in the spectral range of 4000–650 cm^−1^. DTGS was used as a detector.

#### 2.2.3. Differential Scanning Calorimetry Analysis (DSC)

A differential scanning calorimeter (DSC Q200, TA Inc., TA Instruments, New Castle, DE, USA) was used to collect DSC thermograms of the samples and to determine the glass transition temperature of PSAs. About 13–15 mg of dried latex sample was put in a hermetic aluminum pan and was subjected to heating and cooling cycles under nitrogen atmosphere at a specified rate. Equilibration was performed at −85 °C for 5 min, heating at a rate of 5 °C/min to 10 °C, then cooling again to −85 °C at 10 °C/min. After a new equilibration for 5 min at −85 °C, the second heating was carried out at 5 °C/min up to 10 °C.

#### 2.2.4. Thermal Gravimetric Analysis (TGA)

TGA was carried out with a TA Q500 (Waters, TA thermal analysis, Milford, MA, USA). The polymer films (weight around 20 mg) were heated from room temperature to 600 °C at a rate of 10 °C/min under nitrogen atmosphere.

#### 2.2.5. Particle Size and Viscosity

Latex particle sizes were measured using a dynamic light scattering (DLS) instrument (Malvern NanoS Zetasizer, Malvern, UK). The analyses were carried out at 25 °C, and each reported result was an average of three measurements and 16 acquisitions per measurement. The viscosities were measured using a Brookfield Digital Viscosimeter DV-E MODEL (RVDV-E spring torque), AMETEK Brookfield, Middleboro, MA, USA. The analyses were carried out at 25 °C and 40–70% R.H.

#### 2.2.6. Molar Mass Determination

Size-exclusion chromatography multiangle light scattering (SEC-MALS) was carried out with a Malvern Viscotek system, a Triple Detector, and a set of three columns Phenogel Phenomenex 100–105 Å (300 × 4.6 mm, 5 µm); tetrahydrofuran (THF) (HPLC grade, stabilized with BHT, 250 ppm) was used as a carrier solvent (flow rate: 3 mL/min, 30 °C) and an advanced calibration was performed with a narrow polymethyl methacrylate standard. Sample solutions were prepared at room temperature and then filtered on 0.45 µm PTFE filters. The samples were dissolved in THF to achieve a concentration of 3 mg/mL and around 10 µL was injected into the GPC instrument.

#### 2.2.7. Conversion and Solid Content

The conversion was measured by gravimetric analysis via evaporation. About 4 g of latex was weighed into an aluminum foil dish and into a Petri dish. The aluminum foil dish was dried at 75–85 °C; instead, the Petri dish was left for two days at room temperature under a hood. The solid content and the final conversion were calculated using Equations (1) and (2) [34].

(1) $$\mathrm{Solid}\;\mathrm{content}\;(\mathrm{wt}.\%)=\frac{w3-w1}{w2-w1}\times 100,$$

where *w*1 is the weight of the aluminum foil dish, *w*2 and *w*3 are the weights of the latex before and after drying, respectively.

(2) $$\mathrm{Conversion}\;(\mathrm{wt}.\%)=\frac{Solid\;content\;\left(wt.\%\right)\times w4}{w5}\times 100,$$

where *w*4 is the total weight of all the materials added into the glass bottle before polymerization and *w*5 is the total weight of monomers.

#### 2.2.8. Peeling Test

The peel resistance, defined as the force required to remove a tape from a test panel, was evaluated by means of the 180° peel test after 24 h from the tape application. The adhesives were cast on PET film (PET tapes of 200 × 20 mm), then they were applied onto painted sheet metal and cardboard panels. A SUN20 Galdabini tensioner (Galdabini S.p.A., Cardano al Campo, Varese, Italy) was used at a constant speed of 300 mm/min. The analyses were carried out at 25 °C and 40–70% R.H. The average force to detach the tapes was recorded.

## 3. Results and Discussion

As previously written in the Section 2, four syntheses were formulated in this paper. Table 3 summarizes the synthesized latexes with their main properties, including density (g/mL), particle size (nm), viscosity (cP), and other ones present in the table below.

### 3.1. Particle Size and Viscosity

The effects of the reaction time on the particle size and viscosity are shown in Table 3. The latexes synthesized in this study had average particle size values between 109 nm and 145 nm. These are relatively low values, which are probably due to the excessive use of surfactant. The particle size distributions (PSDs) for these latexes were all very narrow and with no apparent secondary peaks with dispersity (Đ) < 0.05 for each different reaction time. A narrow distribution would lead to a higher viscosity; in fact, it is noted that, compared to other works in the literature [37], the copolymers treated here showed higher viscosity values. The viscosity values varied between 4000 and 5000 cP. It was noted that for geraniol-based PSAs, the viscosity increased with the reaction time.

### 3.2. Nuclear Magnetic Resonance

^1^H and ^13^C solution NMR analyses were performed to evaluate the presence of significant amounts of unreacted geraniol in the resulting PSAs, i.e., to confirm that all the employed geraniol was successfully included in the PSA polymeric structure. Indeed, the olefinic protons and carbons of pure geraniol resonate in characteristic frequency ranges (between 5.5 and 5.0 ppm for ^1^H; between 140 and 120 ppm for ^13^C [38]), otherwise unaffected by the signals of the PSA itself. This makes it quite easy to ascertain if residual geraniol is indeed present in the final samples.

**Figure 1:**
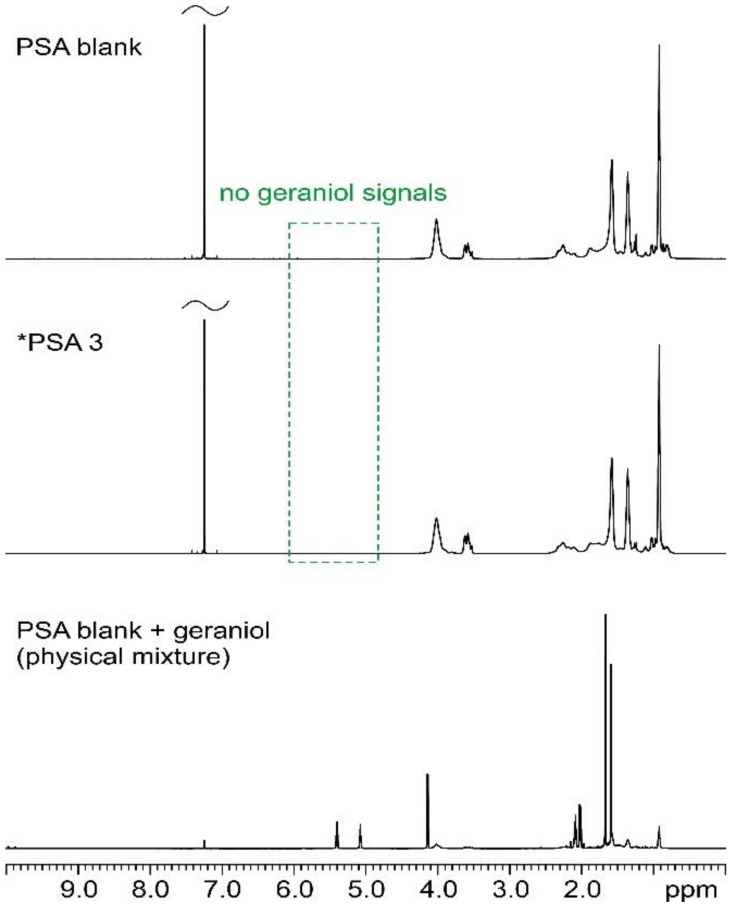
^1^H (600.17 MHz) solution NMR spectra of PSA blank (**top**), *PSA 3 (**center**), and physical mixture (**bottom**) acquired in CDCl_3_ at room temperature. The green dashed box highlights the range of ^1^H chemical shifts in which we would see resonances ascribable to unreacted geraniol, if present.

**Figure 2:**
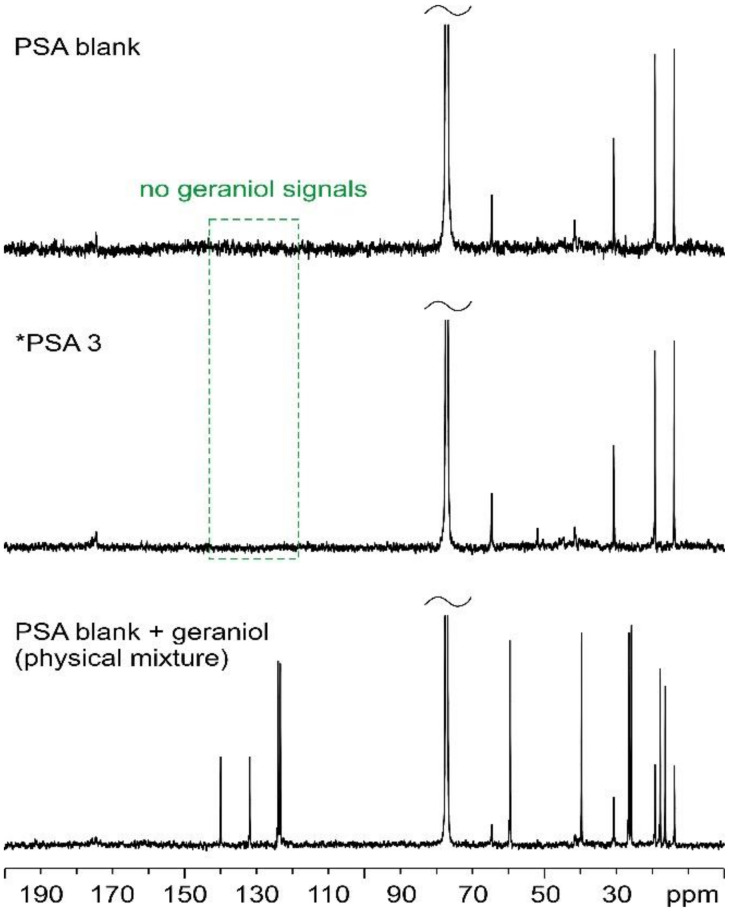
^13^C (150.91 MHz) solution NMR spectra of PSA blank (**top**), *PSA 3 (**center**), and physical mixture (**bottom**), acquired in CDCl_3_ at room temperature. The green dashed box highlights the range of ^13^C chemical shifts in which we would see resonances ascribable to unreacted geraniol, if present.

Figure 1 and Figure 2 show the ^1^H and ^13^C solution NMR spectra, respectively, of PSA blank, *PSA 3, and PSA physical mixture (PSA blank + geraniol) selected as the model samples.

As can be noted, no detectable signals are observed in the chemical shift ranges described above for geraniol in *PSA 3, neither in the ^1^H nor in the ^13^C spectra. In contrast, detectable signals are present in the PSA physical mixture (PSA blank + GER). This provides sufficient evidence to rule out the presence of residual geraniol in *PSA 3.

### 3.3. Fourier-Transform Infrared Spectroscopy (FTIR)

**Figure 3:**
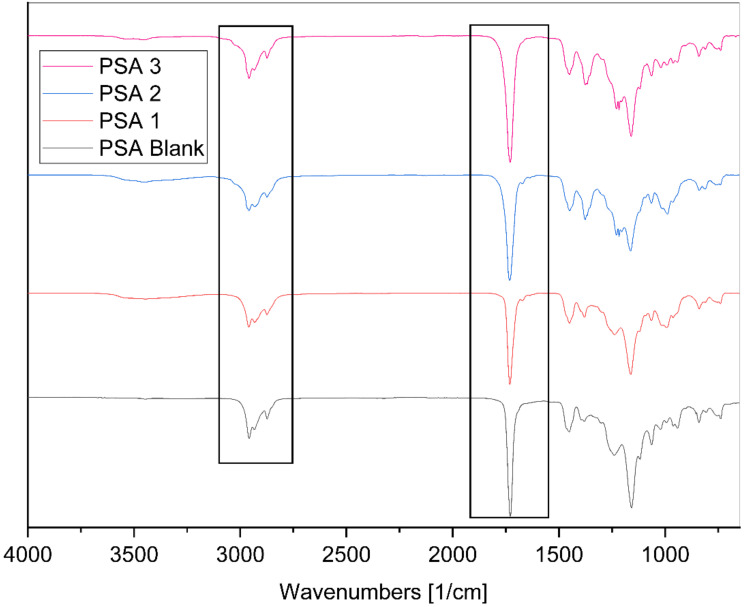
FTIR spectra of reference PSA blank and geraniol-based PSAs.

**Figure 4:**
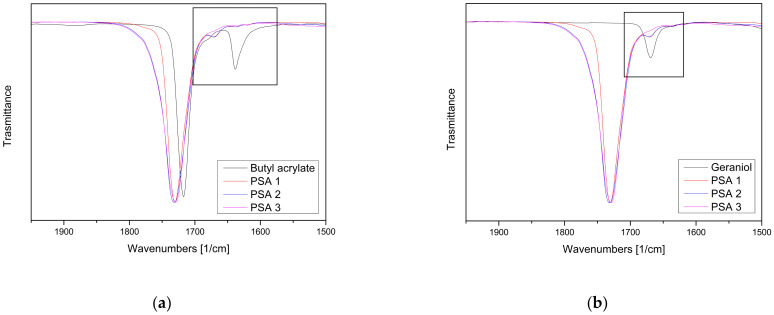
Zoom in the 1900–1500 [1/cm] range of the FTIR spectra of geraniol-based PSAs compared with the FTIR spectra of the main starting monomers (**a**) butyl acrylate and (**b**) geraniol.

To confirm the complete participation of all monomers in the polymerization process, the samples were subjected to FTIR-ATR analyses (see Figure 3). The spectra of the copolymer exhibited prominent bands at 3542 and 3444 cm^−1^, corresponding to the alcoholic groups of geraniol and acrylic acid. The bands ranging from 2959 to 2873 cm^−1^ were attributed to the stretching vibrations of C–H bonds. Notably, at 1730 cm^−1^, a distinctive band characteristic of acrylic polymers emerged, related to the C=O stretching of the ester group. It is crucial to highlight the absence of the peak within the range from 1680 to 1630 cm^−1^, indicative of the C=C stretching in alkenes, a hallmark of the initial monomers (Figure 4b). In Figure 4b, it is important to note that the peak at 1668 cm^−1^, corresponding to the C=C stretching in the geraniol monoterpenes, started to disappear as the reaction time increased. This partial disappearance may suggest that polymerization has occurred between the monomers, providing a significant indication of the reaction taking place. Additionally, the peaks at 1234 cm^−1^ and 1158 cm^−1^ arise from the C–O stretching vibrations of the saturated ester group. The characteristic absorption band of the C–O–C group could be found at 1117 cm^−1^ and 1064 cm^−1^ [39].

### 3.4. Differential Scanning Calorimetry (DSC)

The DSC results of the samples are presented in Table 4. The incorporation of geraniol into the copolymers significantly altered the Tg values. The recorded values indicate a marked decrease in Tg in the geraniol-containing adhesives, from the −31.7 °C of the reference PSA to −53.1 °C of PSA 1. This Tg shift could be attributed to reduced gelation or cross-linking density within the geraniol-modified PSAs, as indicated in previous studies [37]. The reduction in Tg in geraniol-containing PSAs implies an increased mobility of the chains, which is crucial for the necessary anchoring effect in PSA applications. Instead, the trend observed for geraniol adhesives alone is that Tg tends to increase with the reaction time, but not very significantly. The Tg tends to increase by about 1.5 °C for each additional hour of reaction time. The reduction in Tg observed in the PSA containing geraniol can be attributed to the decrease in the cross-linking density and the increased mobility of the polymer chains. This behavior aligns with the observations of Badía et al. (2018), who noted a similar reduction in Tg in bio-based PSAs using geraniol derivatives [12]. It is important to note that the most suitable Tg range for PSAs is typically between −30 and −60 °C. Since the Tg values of geraniol-enhanced PSAs fall within this range, it is plausible to consider geraniol as a valid co-monomer of butyl acrylate (BA) in the formulation of PSAs.

### 3.5. Thermogravimetric Analysis (TGA)

**Figure 5:**
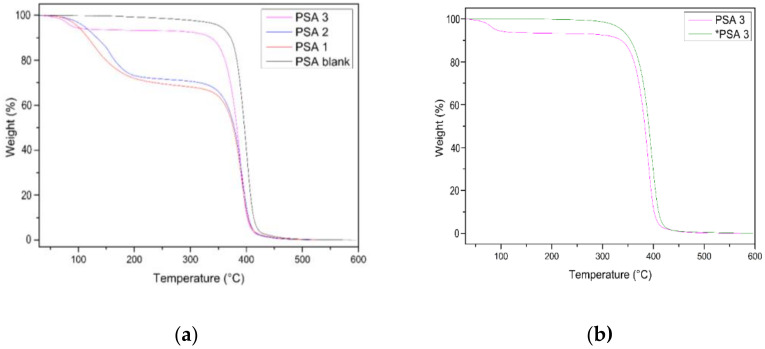
(**a**) TGA for acrylic latex PSA with different reaction times (PSA blank 2.5 h; PSA 1_4h; PSA 2_5 h; PSA 3_6 h). (**b**) TGA of PSA 3 and *PSA 3 with post-polymerization treatment.

In Figure 5, the thermogravimetric analysis (TGA) curves reveal distinct thermal behaviors between the PSA formulations. PSA blank exhibits a single degradation step, which reflects its relatively high thermal stability. In contrast, PSA 1, PSA 2, and PSA 3 display two degradation stages, indicating the influence of geraniol on their thermal properties. Notably, PSA 3 (post-polymerized), following additional treatment, shows only one degradation step, similar to PSA blank, suggesting that post-treatment enhances the thermal stability by eliminating unreacted materials. The data in Table 5 provide further details on these thermal properties. PSA blank has a Tonset of 379 °C and a Tmax of 400 °C, demonstrating its high thermal stability. On the other hand, PSA 1, PSA 2, and PSA 3 show Tonset values of 90 °C, 116 °C, and 118 °C, respectively, which are linked to the evaporation of absorbed water and the decomposition of unreacted components in the first degradation stage. These Tonset values represent the temperatures at which these non-reacted components are lost, before the onset of major polymer degradation. In terms of mass loss, PSA 1 shows a 24% loss, PSA 2 an 18% loss, and PSA 3 a minimal 6% loss in the first stage, suggesting that longer reaction times lead to more complete polymerization. The post-polymerization treatment significantly alters the thermal behavior of PSA 3 (post-polymerized), which shows a Tonset of 358 °C and a Tmax of 399 °C, closely aligning with the thermal behavior of PSA blank. This suggests that the post-polymerization process effectively removes residual volatile components, thereby improving the overall thermal stability of the material. These results are consistent with studies by Worzakowska (2021) [40], which demonstrate that the use of bio-derived monomers, such as geraniol-based ones, can lead to cross-linking efficiency in polymers and thermal degradation resistance comparable to that of petroleum-based PSAs.

### 3.6. Molar Mass Determination

The number average molar mass (Mn), the weight average molar mass (Mw), and the dispersity (Đ) of the latexes are shown in Table 6. It can be immediately seen that, compared to PSA blank, with the introduction of geraniol, the Mw decreased, especially for PSA 1 and PSA 2, and the molar mass distribution (MWD) narrowed slightly. PSA 3 is the geraniol-based acrylic latex that showed the largest Mw. It is possible to say that for geraniol-based PSAs, the Mw increased together with the reaction times. In our study, the dispersity (Đ) values were comparable to those reported for acrylic PSAs, where Đ typically ranges between 1.1 and 2.5, depending on the polymerization conditions and use of bio-based monomers. For instance, studies have shown that incorporating terpene-derived monomers, such as geraniol, can help control molecular weight distribution, potentially acting as a chain transfer agent, as observed in PLA formulations [41].

### 3.7. Peeling Test

**Figure 6:**
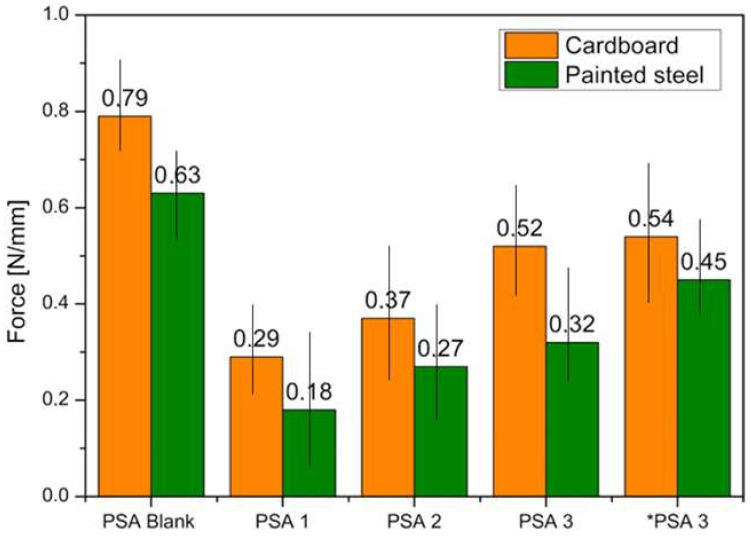
A 180° peeling test of the acrylic latex PSAs.

The adhesion properties of water-based copolymers containing geraniol were determined, as reported in Figure 6. It is important to note that no effort was made to optimize the adhesion properties of these PSAs. The primary objective of this study was to demonstrate the feasibility of synthesizing these materials using geraniol as the monomer, without any functionalization. To evaluate the adhesive properties, the dispersions were applied to two different substrates: cardboard and painted steel. After thorough drying, a transparent and uniform polymer film was observed. Peel strength was evaluated according to the procedures described in the Section 2. The effect of the reaction time on the peel strength is shown in Figure 6. For the reference, PSA blank, values equal to 0.79 N/mm on cardboard and 0.63 N/mm on painted steel were observed. For the green PSAs containing geraniol, on cardboard substrate, the following adhesion values were recorded: PSA 1 (0.29 N/mm), PSA 2 (0.37 N/mm), PSA 3 (0.52 N/mm), and *PSA 3 (0.54 N/mm). For the green PSAs containing geraniol, on the steel-painted substrate, the following adhesion values were recorded: PSA 1 (0.18 N/mm), PSA 2 (0.27 N/mm), PSA 3 (0.32 N/mm), and *PSA 3 (0.45 N/mm). It was found that all the petroleum- and geraniol-based adhesive copolymers showed better performances on the cardboard substrate. Among the synthesized formulations, as expected, the best performance was observed for the reference PSA blank. The peel strength values (0.52 N/mm on cardboard) are lower compared to the 0.79 N/mm achieved with petroleum-derived PSAs but exceed the 0.29 N/mm reported for terpene-based adhesives by Maassen et al. (2016) [17]. *PSA 3 showed the best adhesive performance among the geraniol-based PSAs. From the data shown, it can be seen that the bond strength increases with the increasing reaction time for both analyzed substrates. It is well known that the adhesive strength generally decreases with the increasing Tg. However, in this case, the increase in Tg was marginal. The performance of an acrylic PSA can vary significantly depending on the substrate to which it is applied, due to differences in the surface energy, consistency, and chemical compatibility. Cardboard is a porous, fibrous material with a relatively high surface energy. Acrylic PSAs can penetrate the surface and form a mechanical bond with the fibers. This can certainly provide strong adhesion, but performance can also be affected by other factors such as surface roughness, absorbency, and surface treatments. Due to the uneven surface of the board, areas of poor contact are created; additionally, the porous nature can lead to absorbing the PSA, weakening adhesion. In addition, some boards are surface-treated or coated to resist moisture, which can affect adhesion as well [42,43].

## 4. Conclusions

In this study, the potential of geraniol, a 100% bio-based monomer, was investigated as a sustainable alternative to petroleum-derived butyl acrylate in the formulation of water-based acrylic pressure-sensitive adhesives (PSAs). Through a semi-continuous emulsion polymerization process, a series of latexes with varying reaction times were synthesized, with the goal of identifying the optimal conditions for incorporating geraniol into acrylic PSAs without chemical modification. The results demonstrate that geraniol can effectively replace a significant portion of butyl acrylate in PSA formulations, achieving attractive adhesive performances while improving environmental sustainability. PSA 3, synthesized with a reaction time of 6 h, exhibited the most favorable properties, with a glass transition temperature (Tg) of −49.2 °C, which is within the ideal range for effective PSAs. This formulation showed excellent thermal stability, comparable to that of conventional PSAs, with a weight loss in the first degradation step of only 6%. It also exhibited a high level of monomer conversion and effective polymerization. In addition, molecular weight analysis revealed that PSA 3 possesses a higher molecular weight than other geraniol-enhanced formulations, suggesting a more robust polymer structure. Peeling tests further confirmed the practical applicability of the geraniol-containing PSAs, particularly PSA 3, which demonstrated superior adhesive performance after post-synthesis methanol treatment, highlighting the potential for further optimization. These results underscore the feasibility of using geraniol as a sustainable co-monomer in acrylic PSAs, offering a promising pathway to reduce the dependence of the adhesives industry on fossil-based resources. This research not only aligns with global sustainability goals, but also opens new avenues for the development of environmentally friendly adhesive products.

Future work will focus on optimizing these formulations, evaluating the introduction of additives to improve adhesive performance, exploring the scalability of the production process, and assessing the long-term environmental impact of geraniol-based PSAs in real-world applications. The obtained compositions show the potential for commercialization; in the future, they can be used as a base in the adhesives and self-adhesive materials industry for obtaining acrylate tapes and self-adhesive materials meeting the principles of “5E”, which are efficiency, energy saving, enabling, economy, and environmental friendly.

## Figures and Tables

**Table 1:** Summary of the synthesized PSA latexes *.

Synthesized PSA Latexes	Formulation (wt.% Monomers)	Reaction Time (h)
PSA blank	BA:MMA:AA:HEMA (87:10:1:2)	2.5
PSA 1	BA:GER:MMA:AA:HEMA (67:20:10:1:2)	4
PSA 2	BA:GER:MMA:AA:HEMA (67:20:10:1:2)	5
PSA 3	BA:GER:MMA:AA:HEMA (67:20:10:1:2)	6

* This table does not include the *PSA 3 copolymer because the monomer ratios and reaction times are identical to those of PSA 3, while the post-polymerization purification was different.

**Table 2:** Amounts employed in the synthesis of the acrylic latex PSA.

Role/Function	Constituent	Acronyms	Weight (g)
^1^ Soft monomer	Butyl acrylate	BA	67–87
^2^ Soft monomer	Geraniol	GER	0–20
Hard monomer	Methyl methacrylate	MMA	10
Functional monomer	Acrylic acid	AA	1
	Hydroxy ethyl methacrylate	HEMA	2
Emulsifier	Sodium dodecyl sulfate	SDS	1.5
Initiator	Potassium persulfate	KPS	0.7
Buffer	Sodium bicarbonate	NaHCO_3_	0.15
Continuous phase	Water	H_2_O	95

^1^ A 100% petroleum-based soft monomer. ^2^ A 100% bio-based 100% soft monomer.

**Table 3:** Summary and characteristics of the synthesized PSA latexes.

PSA ID	Density (g/mL)	Solid Content	Conversion (%)	Viscosity (cP)	Particle Size (nm)	Đ
PSA blank	1.06	55%	97%	5000	145.7	0.043
PSA 1	0.96	52%	73%	4000	108.9	0.044
PSA 2	0.93	52%	81%	4300	115.6	0.045
PSA 3	1.12	52%	94%	4800	121.9	0.043

**Table 4:** Glass transition (Tg) values, obtained by DSC analysis, of formulated PSAs.

PSA ID	Tg (°C)
PSA blank	−31
PSA 1_4 h	−53
PSA 2_5 h	−51
PSA 3_6 h	−49
*PSA 3_6 h	−48

*PSA 3 refers to a purified version of the original PSA 3, which underwent a purification process wherein the copolymer was precipitated from the aqueous phase by the addition of methanol.

**Table 5:** TGA for acrylic PSA with different reaction times. b Weight change is referred to the percentage of weight loss at the end of the first step.

PSA ID	Weight Change ^a^	T_onset_ (1%) (°C)	T_max_ (°C) ^b^	Residual Mass (%) @ 600 °C
PSA blank	-	379 °C	400	3.9
PSA 1_4 h	24%	90 °C	388	7.2
PSA 2_5 h	18%	116 °C	393	3.2
PSA 3_6 h	6%	118 °C	399	6.8
*PSA 3_6 h	-	358 °C	399	6.8

^a^ Weight change is referred to the percentage of weight loss at the end of the first step. ^b^ For PSA 1, PSA 2, and PSA 3, where two mass losses are observed in the thermogram, the Tmax refers to the second mass loss.

**Table 6:** Mw measurements for acrylic PSAs with different reaction times.

	Reaction Time	GER Amount (wt.%)	Mw (g/mol)	Mn (g/mol)	Đ
PSA blank	2.5 h	20	98,806.00	52,241	1.77
PSA 1	4 h	20	44,318.00	22,052	2
PSA 2	5 h	20	68,699.00	39,207	1.75
PSA 3	6 h	2	83,415.00	42,829	1.7

## Data Availability

The original contributions presented in the study are included in the article/Supplementary Materials, further inquiries can be directed to the corresponding author.

## Supplementary Materials

The following supporting information can be downloaded at: https://www.mdpi.com/article/10.3390/ma17204957/s1, Figure S1: The curves of molecular weight distributions with different reaction time of copolymer: (a) PSA blank (reference) and (b) PSAs with geraniol; Figure S2: The 13C NMR spectrum of PSA 2 shows a peak at 125 ppm, corresponding to the double bonds of geraniol; Figure S3: Image of some adhesive detachment; Figure S4: DTG for acrylic latex PSA with different reaction time (PSA blank 2.5 h; PSA 1 4 h; PSA 2 5 h; PSA 3 6 h and *PSA 3 with post-polymerization treatment); Table S1: Cohesive test at 20 °C and 70 °C and SAFT test (the adhesives copolymers don’t contain crosslinker agent.
