# Supplementary material for: Advancing Sustainability: Geraniol-Enhanced Waterborne Acrylic Pressure-Sensitive Adhesives without Chemical Modification
Source: Materials (Basel). 2024 Oct 10;17(20):4957. doi: 10.3390/ma17204957 (PMC11509658; doi:10.3390/ma17204957)
Supplement: Supplementary file 1 [file materials-17-04957-s001.zip › materials-3219253-supplementary.pdf]

Supplementary Materials

(a)

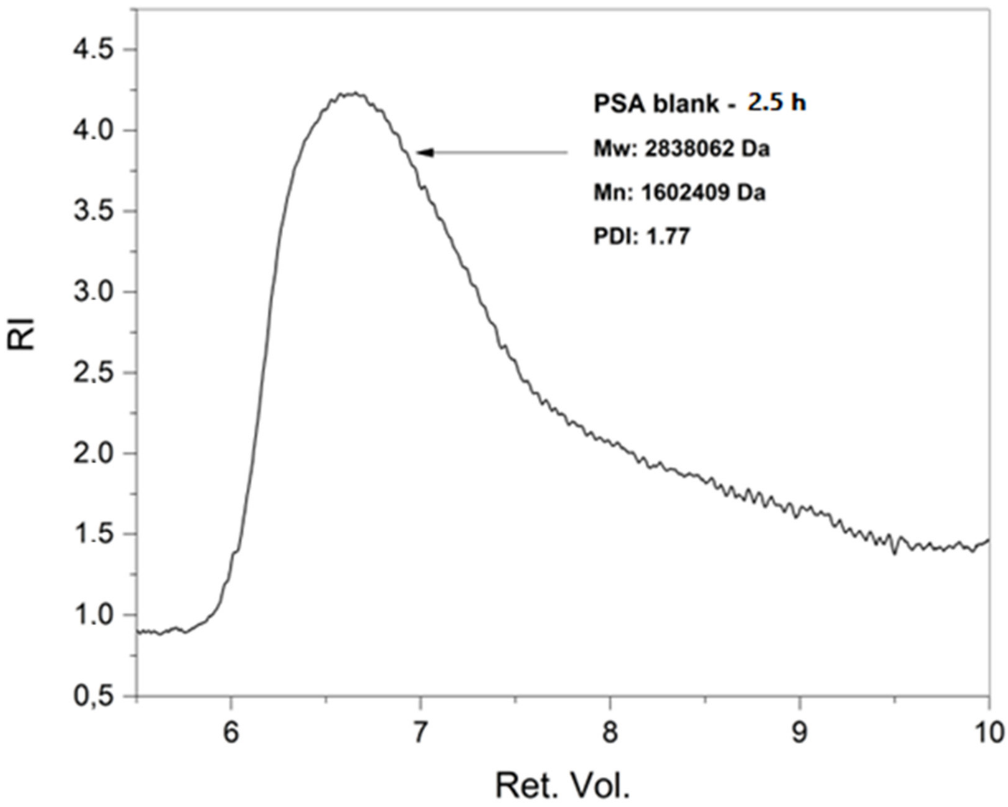

(b)

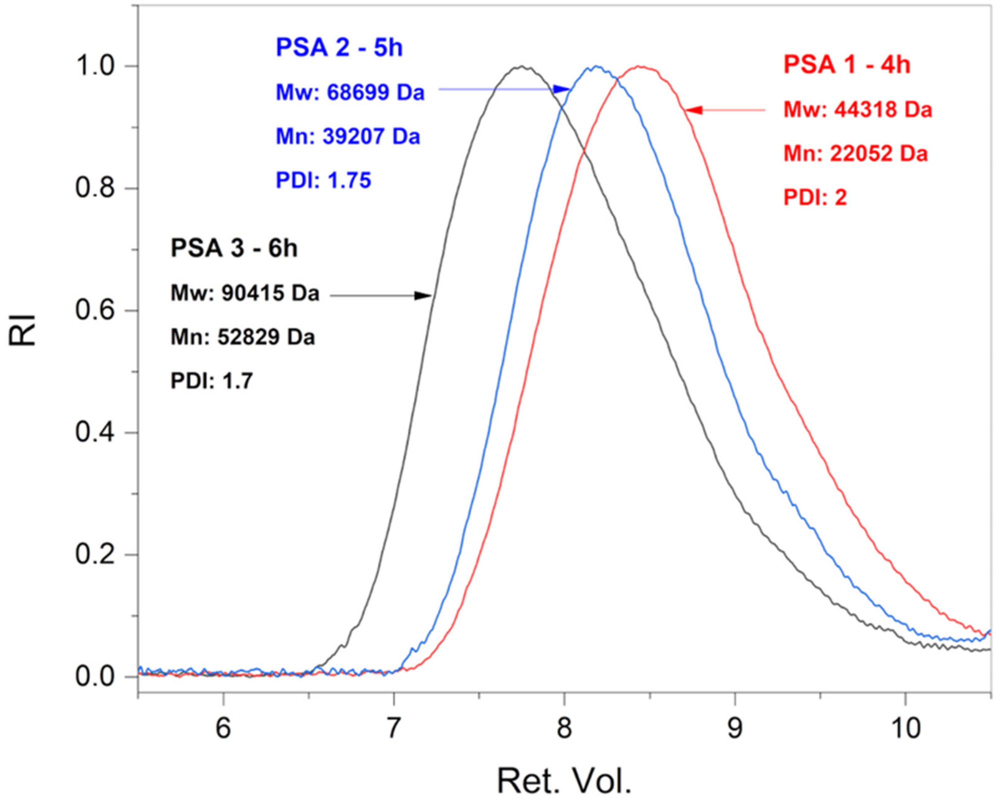

Figure S1 The curves of molecular weight distributions with different reaction time of copolymer: (a) PSA blank (reference) and (b) PSAs with geraniol.

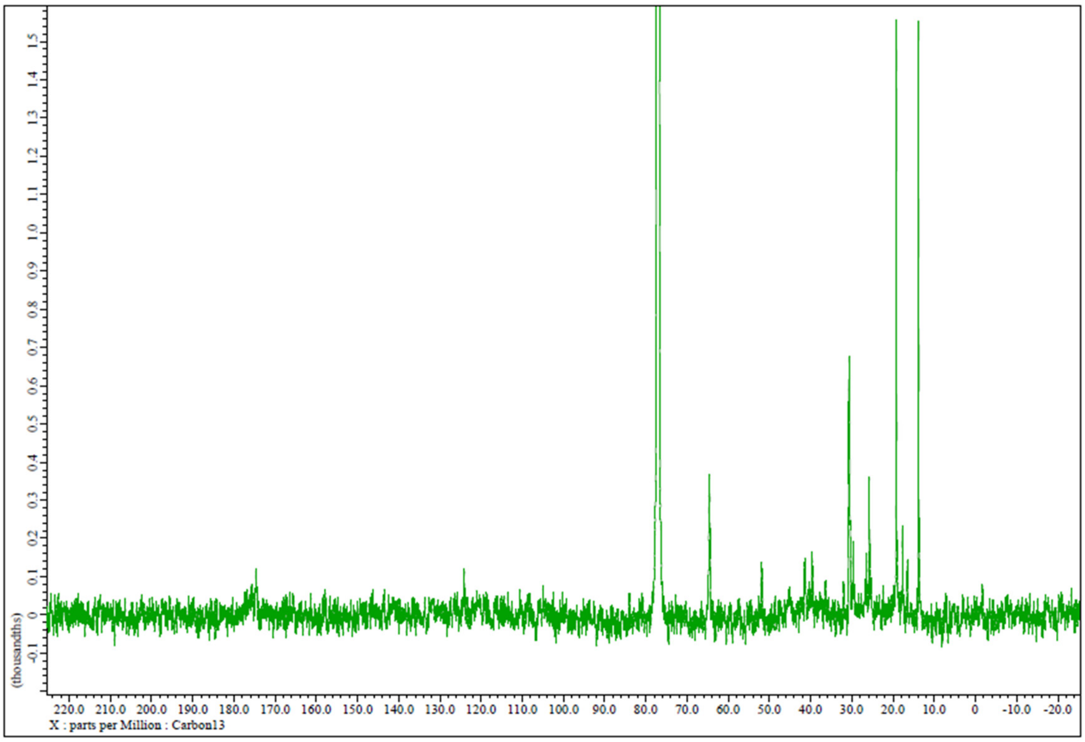

Figure S2: The 13C NMR spectrum of PSA 2 shows a peak at 125 ppm, corresponding to the double bonds of geraniol.

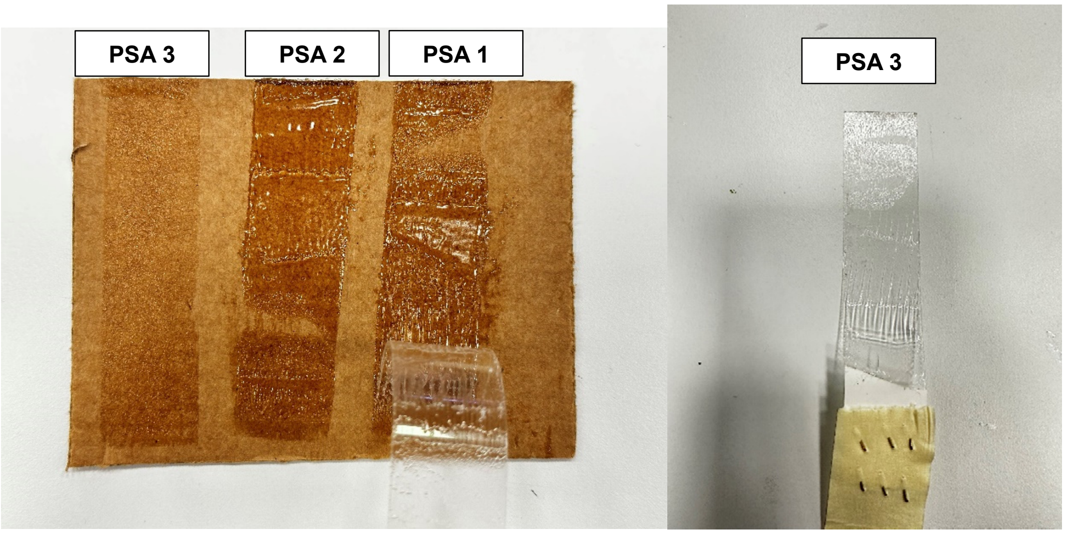

Figure S3: Image of some adhesive detachment

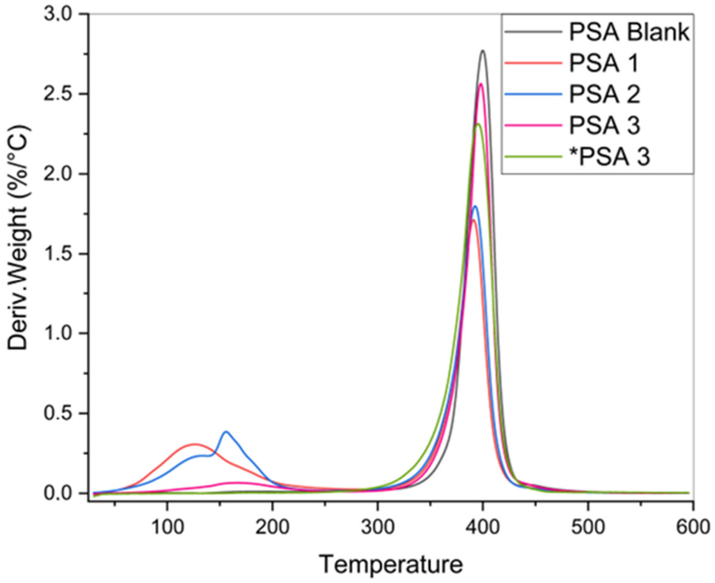

Figure S4: DTG for acrylic latex PSA with different reaction time (PSA blank 2.5 h; PSA 1 4 h; PSA 2 5 h; PSA 3 6h and *PSA 3 with post-polymerization treatment).

Table S1: Cohesive test at 20 °C and 70 °C and SAFT test (the adhesives copolymers don't contain crosslinker agent

	**Cohesion [h]**	**Cohesion [h]**	**SAFT**
	**20 °C**	**70 °C**	**(°C)**
PSA Blank	>72	>72	203
PSA 1	< 1	<1	26
PSA 2	< 1	<1	32
PSA 3	< 1	<1	30
